# Uptake of WHO Recommendations for First-Line Antiretroviral Therapy in Kenya, Uganda, and Zambia

**DOI:** 10.1371/journal.pone.0120350

**Published:** 2015-03-25

**Authors:** Herbert C. Duber, Emily Dansereau, Samuel H. Masters, Jane Achan, Roy Burstein, Brendan DeCenso, Anne Gasasira, Gloria Ikilezi, Caroline Kisia, Felix Masiye, Pamela Njuguna, Thomas Odeny, Emelda Okiro, D. Allen Roberts, Emmanuela Gakidou

**Affiliations:** 1 Institute for Health Metrics and Evaluation, University of Washington, Seattle, WA, United States of America; 2 Infectious Disease Research Collaboration, Makerere University, Kampala, Uganda; 3 Action Africa Help-International, Nairobi, Kenya; 4 Department of Economics, University of Zambia, Lusaka, Zambia; University of Athens, Medical School, GREECE

## Abstract

**Introduction:**

Antiretroviral therapy (ART) guidelines were significantly changed by the World Health Organization in 2010. It is largely unknown to what extent these guidelines were adopted into clinical practice.

**Methods:**

This was a retrospective observational analysis of first-line ART regimens in a sample of health facilities providing ART in Kenya, Uganda, and Zambia between 2007-2008 and 2011-2012. Data were analyzed for changes in regimen over time and assessed for key patient- and facility-level determinants of tenofovir (TDF) utilization in Kenya and Uganda using a mixed effects model.

**Results:**

Data were obtained from 29,507 patients from 146 facilities. The overall percentage of patients initiated on TDF-based therapy increased between 2007-2008 and 2011-2012 from 3% to 37% in Kenya, 2% to 34% in Uganda, and 64% to 87% in Zambia. A simultaneous decrease in stavudine (d4T) utilization was also noted, but its use was not eliminated, and there remained significant variation in facility prescribing patterns. For patients initiating ART in 2011-2012, we found increased odds of TDF use with more advanced disease at initiation in both Kenya (odds ratio [OR]: 2.78; 95% confidence interval [CI]: 1.73-4.48) and Uganda (OR: 2.15; 95% CI: 1.46-3.17). Having a CD4 test performed at initiation was also a significant predictor in Uganda (OR: 1.43; 95% CI: 1.16-1.76). No facility-level determinants of TDF utilization were seen in Kenya, but private facilities (OR: 2.86; 95% CI: 1.45-5.66) and those employing a doctor (OR: 2.86; 95% CI: 1.48-5.51) were more likely to initiate patients on TDF in Uganda.

**Discussion:**

d4T-based ART has largely been phased out over the study period. However, significant in-country and cross-country variation exists. Among the most recently initiated patients, those with more advanced disease at initiation were most likely to start TDF-based treatment. No facility-level determinants were consistent across countries to explain the observed facility-level variation.

## Introduction

Since 2006, the World Health Organization (WHO) has encouraged countries to transition away from first-line antiretroviral therapies (ART) containing stavudine (d4T), due to well-recognized toxicities including lactic acidosis, lipodystrophy, and peripheral neuropathy.[1–4] The 2010 WHO guidelines solidified this recommendation, stating that “countries should take steps to progressively reduce the use of d4T in first-line regimens.”[5–7] In lieu of d4T, the WHO and other expert bodies recommended first-line adult ART that utilized a nucleoside reverse transcriptase inhibitor (NRTI) backbone of either zidovudine (AZT) or tenofovir (TDF).[5][8]

While numerous studies comparing d4T-, TDF- and AZT-based regimens have found no significant differences in efficacy and viral load suppression, patients initiated on d4T-based treatment are more likely to require ART substitution due to drug toxicity. [9–13] In turn, regimen switching may increase the likelihood of drug resistance.[14] TDF combined therapy offers an additional benefit beyond both d4T and AZT, as it can be taken as a single daily dose, which may portend improved treatment adherence.[13] Based on this advantage, the 2013 WHO consolidated guidelines on the use of ART named a TDF-based therapy as the preferred option with AZT-based therapies listed as alternatives.

However, AZT- and TDF-based regimens are more expensive than d4T. For instance, under prices negotiated by the Clinton Health Access Initiative (CHAI) in 2010, triple combination therapies containing AZT and TDF were at least 80% and 250% more expensive than those based on d4T, respectively.[15] Since then, CHAI has successfully negotiated substantially lower prices for AZT and TDF combination therapy, though in 2013 they were still at least 40% and 66% more expensive than d4T-based therapies, respectively. As a result, there was and continues to be concern that low-income countries hardest hit by the HIV/AIDS epidemic, as well as donors who supply much of the ART medication, would be hesitant to follow these new recommendations due to cost.[9][10] Despite cost concerns, many countries in regions of high HIV/AIDS burden, including Kenya, Uganda, and Zambia, adopted national guidelines recommending phasing out d4T in favor of either AZT or the more expensive TDF.[16–18]

In this study we examine if WHO and national guidelines to phase out d4T use were adopted into practice in Kenya, Uganda, and Zambia and the pace at which they were adopted at the health facility level. We additionally examine the relative uptake of AZT- and TDF-based therapies, and examine the patient- and facility-level factors associated with the prescription of TDF-based ART in 2011–2012 in Kenya and Uganda.

## Methods

### Facility selection

This study was performed on a subcomponent of facilities selected for a larger facility-based costing study in Kenya, Uganda, and Zambia. Data were collected between November 2011 and November 2012. In each country, health districts were first stratified by a variety of indicators including wealth, health, and population characteristics. A matrix of possible combinations was then developed, and at least one district from each populated combination was chosen for inclusion in our study sample. In all countries there was purposeful inclusion of the capital cities: Nairobi, Kampala, and Lusaka. The following links provide a detailed description of the study protocol (http://www.healthdata.org/sites/default/files/files/Projects/DCPN/ABCE%20Project%20Cross-Country%20Protocol.pdf), as well as country-specific policy reports for the broader facility-based costing study (http://ghdx.healthdata.org/series/access-bottlenecks-costs-and-equity-abce-project).

The selection of facilities within each district varied by country, but in all cases we sampled a range of hospitals and health centers, both rural and urban. Health facilities that declined study participation or where access to the facility was limited due to safety, travel distance, or time constraints were replaced with other similar facilities within the same district by the country team when a suitable replacement facility was identified. A subset of facilities that provided ART services were then selected for inclusion in the ART component of the study described below.

### Facility survey

Six individual survey modules were implemented, collecting information on each facility’s finances, management, medical consumables, equipment, capacity, services, drugs, and outputs. This information was supplemented by information obtained from the District Health Management Team where applicable.

### Patient chart selection and record extraction

At each of the designated ART facilities that agreed to patient chart extraction, research assistants asked to see all patient records, specifically requesting charts of deceased patients and those lost to follow-up, in addition to patients currently enrolled in treatment. Patient charts were eligible for record extraction if the patient was (or would have been) an adult (age 18 and older) at the time of extraction, and he/she initiated ART between six and 60 months prior to the date of chart extraction. Once all available and eligible outpatient records were identified, the research assistant either estimated the total number of charts with the help of the facility administrator, or the number of charts, if known, was recorded. In facilities in which there were fewer than 250 charts, all charts were extracted. If a facility had more than 250 charts, the total number of charts was divided by 250 and then using the closest integer (*x*) to that result, every *x* chart was selected for record extraction until 250 charts were reviewed.

Data extracted from the patient record included dates related to pre-ART enrollment, ART initiation, and clinic visits. Demographic characteristics, clinical information (including laboratory testing, WHO staging, and weight), and ART regimen were also recorded.

### Data management and analysis

Data were collected electronically using Datstat Illume Survey Manager 5.1 (DatStat Inc., Seattle, WA). All information was then uploaded and analyzed using STATA version 13.0 (StataCorp, College Station, Texas).

We excluded charts from facilities that failed to meet a quota of 50 total charts. In addition, individual facility-years with fewer than 10 patient charts were excluded from descriptive analyses to prevent a small number of charts from exerting excessive influence in a given facility-year. In addition, patients lacking information about initial regimen, who initiated before turning 18 years of age, or initiated outside our study period (2008–2012 for Uganda and Kenya and 2007–2011 for Zambia, based on the timing of data collection) were also excluded. In cases where WHO stage at initiation was missing, “missing stage at initiation” was included as a category in the regression rather than excluding the patient from the analysis.

Antiretroviral treatment regimen at the time of initiation is presented by NRTI backbone (d4T, AZT, TDF, or other). Patient charts within a facility are classified by the year of ART initiation. Charts are weighted based on the size of the ART program in the year of ART initiation as obtained from the facility survey. The total number of adult initiates at a facility was missing for approximately 5% of facility-years and was linearly interpolated based on existing data points for that facility. Additionally, values were linearly extrapolated for the most recent year for all facilities because facility data was only collected through 2010 in Zambia and 2011 in Kenya and Uganda.

A mixed effects logistic regression model was used to evaluate for both patient and facility predictors of TDF-based treatment for patients initiating ART in 2011–2012. Separate models were run for Kenya and Uganda, while Zambia was not included in this part of the analysis due to lack of variation in regimens for the most recent patient cohort. The logistic regressions were run at the patient level, with a binary outcome equal to 1 if the patient was initiated on a TDF-based regimen and 0 if they were initiated on any other regimen. Independent patient variables included sex, initiation year, age at initiation, WHO stage at initiation, and whether they had a baseline CD4 test. Independent facility variables included ownership, level of complexity (hospital versus clinic), location, program age, presence of a physician, whether nurses initiated patients at the facility, and whether any HIV training had occurred at the facility in the year prior to the survey. Random facility intercepts were also included.

### Ethical considerations

Ethical approval for this study was obtained from the University of Washington Human Subjects Division as well as local institutional review boards in Kenya (Kenya Medical Research Institute Ethics Review Committee), Uganda (Makerere University School of Medicine Research Ethics Committee), and Zambia (The University of Zambia Biomedical Research Ethics Committee). Patient consent for review of medical records was not obtained, as all information extracted from clinical charts was anonymized and de-identified prior to analysis.

### Role of the funding source

The sponsor had no role in the study design, data collection, data analysis, data interpretation, or writing of the report. The corresponding author had full access to all the data in the study and had final responsibility for the decision to submit for publication.

## Results

We extracted a total of 33,490 charts from 160 health facilities providing ART services in Kenya, Uganda, and Zambia. After applying the general exclusion criteria, our analytical dataset contained 29,507 charts from 146 facilities (13,135 from 51 facilities in Kenya, 8,097 from 47 facilities in Uganda, and 8,275 from 48 facilities in Zambia). Less than 5% of eligible charts were excluded from descriptive analyses because they initiated in facility-years with insufficient observations or transferred from a different facility. 5.7% of charts were excluded from the regression analyses because they were missing information on one or more of the covariates.

Three-quarters of facilities surveyed were managed by the government, 66% were in urban areas, and 54% were hospitals (Table 1). The median ART program was six years old at the time of the survey. The resources and practices surrounding ARV acquisition varied by country. At the time of the facility survey, most facilities in Kenya (98.0%) and Uganda (87.2%) determined the quantity of ARVs ordered internally, compared to 7.1% in Zambia. Nearly all Kenyan facilities (98.0%) carried a fixed-dose triple combined form of TDF, compared to less than half of Uganda facilities (48.9%). Approximately a quarter of facilities in Kenya and Uganda had experienced a TDF stockout in the quarter preceding the survey. In terms of staffing, Ugandan facilities were most likely to have a physician on the ART staff (68.1%) and least likely to allow nurses to initiate patients (19.1%). More facilities in Uganda (36.2%) than Kenya (15.7%) reported training staff on HIV in the past year.

**Table 1:** Facility characteristics at time of survey.

	Kenya	Uganda	Zambia	Total
N	51	47	48	146
	%	%	%	%
Facility owner				
Government	90.2	66	68.8	75.3
Private/NGO	9.8	34	31.3	24.7
Complexity				
Non-hospital	47.1	38.3	52.1	45.9
Hospital	52.9	61.7	47.9	54.1
Location				
Urban	70.6	66	62.5	66.4
Rural	29.4	34	37.5	33.6
ART program age (median (IQR))	6 (5–8)	7 (5–8)	6 (4–7)	6 (5–7)
Over 5 years	37.3	26.7	38.1	34.1
5 years or less	62.7	73.3	61.9	65.9
ART program has physician	20.8	68.1	42.2	43.6
ARV order quantity is decided internally	98.0	87.2	7.1	66.7
Nurses can initiate patients	34.7	19.1	62.8	38.1
Staff received HIV training in last year	15.7	36.2	.	25.5
Experienced TDF stockout in last quarter	25.5	25.5	.	25.5
Carried TDF in triple dose fixed form	98.0	48.9	.	74.0

The patient sample was 63% female with a median age of 35 years (Table 2). Over a quarter of patients were missing a baseline CD4 value, and 8% were missing a baseline WHO stage. Among those with recorded values, the median CD4 count was 173. Patients most commonly initiated at WHO stage III (37.8%) or II (33.2%), with less than 6% initiating with stage IV disease.

**Table 2:** Patient characteristics at initiation.

	Kenya	Uganda	Zambia	Total
N	13,135	8,097	8,275	29,507
	%	%	%	%
Female	65.9	61.4	58.5	62.6
Age (median (IQR))	35 (28–43)	35 (29–42)	36 (30–42)	35 (29–42)
18–29	28.9	27.6	23.8	27.1
30–39	37.6	39.3	43.7	39.8
40–49	22.3	22.6	22.7	22.5
50+	11.3	10.6	9.8	10.7
WHO stage				
I	20.4	20.1	24.5	21.5
II	32.5	34.7	23.4	30.6
III	33.6	29	42.3	34.8
IV	3.7	6.3	6.4	5.2
Missing	9.7	9.8	3.5	8
CD4 count (median(IQR))	173 (79–259)	175 (83–264)	170 (96–256)	173 (86–259)
<50	11.8	13	9	11.4
50–199	27.8	28.6	41.4	31.8
200–349	23.3	25.3	27.3	25
350+	5.6	6.8	6.4	6.2
Missing	31.5	26.2	16	25.7
Year				
2007			14.1	3.9
2008	18.6	11.8	18.9	16.8
2009	20	16.4	27.3	21
2010	25.9	23.1	35.5	27.8
2011	29.4	33.3	4.3	23.4
2012	6.1	15.5		7

**Fig 1:**
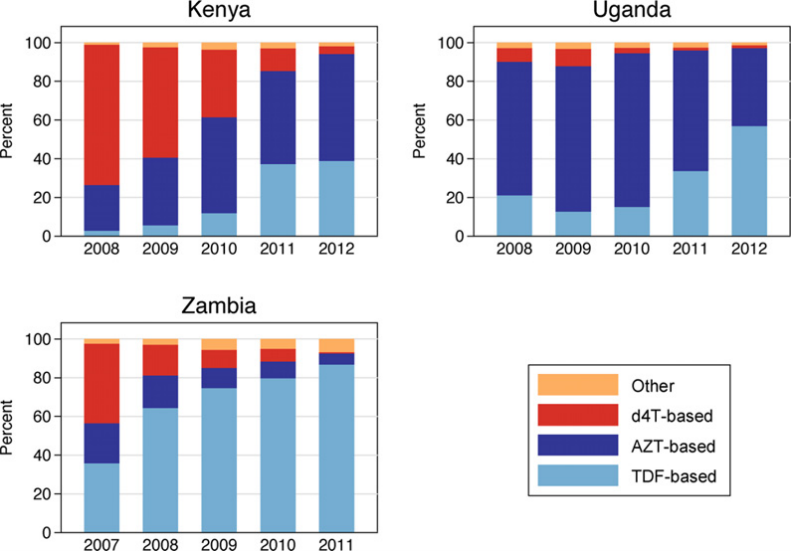
Percentage of patients on d4T-, TDF-, and AZT-based ART regimens by year of treatment initiation.

Fig. 1 illustrates the initial antiretroviral treatment regimens prescribed during our study period. Kenya had the highest proportion of patients prescribed d4T-based regimens in 2008 with 73%, followed by Zambia (16%) and Uganda (7%). By 2011, the proportion of patients initiated on d4T-based regimens had dropped to 12% in Kenya and 1% in Zambia and Uganda. TDF use rose during the same period, from 3% to 37% in Kenya, 2% to 34% in Uganda, and 64% to 87% in Zambia. Zambian patients were rarely prescribed AZT (6%) in 2011, while it was common in Uganda (62%) and Kenya (48%).

**Fig 2:**
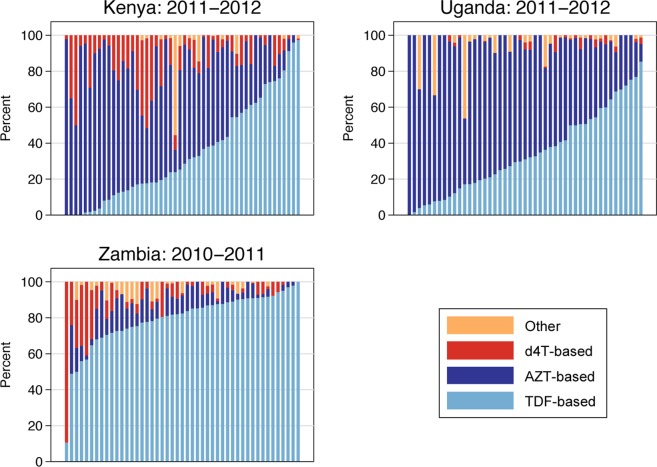
Percentage of patients on d4T-, TDF-, and AZT-based ART regimens by facility in the most recent years of data available.

At the health facility level, we observed variation both within and between countries with regard to the ART regimen at initiation prescribed in a given year. In 2008, the median percentage of patients initiated on d4T-based regimens at a given facility was 85% (interquartile range [IQR]: 76%-94%) in Kenya, 13% (IQR: 5%-22%) in Uganda, and 9% (IQR: 5%-22%) in Zambia. Fig. 2 demonstrates the degree of facility-level variation in the most recent patient cohort for each country. The median percentage of patients initiated on d4T at a given facility in 2011–2012 in Kenya and Uganda and in 2010–2011 in Zambia was 7% (IQR: 3%-17%), 0% (IQR:0%-2%), and 4% (IQR: 2%-9%), respectively. Facility-level rates of TDF use in the same years of initiation were 27% (IQR: 13%-57%) in Kenya, 31% (IQR: 17%-51%) in Uganda, and 83% (IQR: 73%-90%) in Zambia.

The regression analysis using the most recent data from Kenya and Uganda found that initiating in 2012 versus 2011 was significantly associated with a higher probability of receiving TDF in both countries (Table 3). Patients with a more advanced WHO stage at initiation were also significantly more likely to begin a TDF-based therapy; compared to patients classified as WHO stage I, those classified as stage IV had 2.78 (95% confidence interval [CI]: 1.73–4.48) and 2.15 (95% CI: 1.46–3.17) times the odds of initiating on TDF in Kenya and Uganda, respectively. Having a CD4 test at initiation (regardless of test result) was also a significant predictor in Uganda (odds ratio [OR]: 1.43; 95% CI: 1.16–1.76). Finally, several facility characteristics were significantly associated with TDF use in Uganda, including employing a doctor for the ART program (OR: 2.86; 95% CI: 1.48–5.51) and having private or non-governmental organization owner (OR: 2.86; 95% CI: 1.45–5.66).

**Table 3:** Multilevel logistic regression analysis for odds of initiation on TDF-based therapy in 2011–2012.

	Kenya		Uganda	
	OR	[95% CI]	OR	[95% CI]
Facility characteristics				
Private/NGO owner	0.35	[0.03,3.81]	2.86	[1.45,5.66]**
Hospital	2.83	[0.71,11.22]	0.61	[0.27,1.38]
Rural	0.99	[0.25,3.98]	0.63	[0.32,1.26]
Program is over 5 years old	0.45	[0.15,1.41]	1.93	[0.92,4.02]
ART program has physician	2.29	[0.51,10.35]	2.86	[1.48,5.51]**
Nurses can initiate patients	0.54	[0.19,1.57]	1.42	[0.58,3.44]
Staff received HIV training in last year	1.41	[0.29,6.76]	0.8	[0.42,1.52]
Patient characteristics				
Female	1.15	[0.98,1.34]	0.86	[0.73,1.02]
CD4 recorded at initiation	1.16	[0.97,1.39]	1.43	[1.16,1.76]**
Year of initiation (2012 vs 2011)	1.59	[1.30,1.96]**	2.53	[2.07,3.09]**
Age at initiation (reference: under 30)				
30–40	1.29	[1.07,1.55]**	1.02	[0.83,1.24]
40–50	1.18	[0.95,1.47]	1.04	[0.83,1.32]
50+	1.39	[1.07,1.79]*	1.31	[0.98,1.74]
WHO stage at initiation (reference: Stage 1)				
2	1.13	[0.92,1.38]	0.88	[0.70,1.10]
3	2.14	[1.74,2.63]**	1.28	[1.00,1.64]
4	2.78	[1.73,4.48]**	2.15	[1.46,3.17]**
Missing	1.09	[0.77,1.54]	1.24	[0.90,1.71]

*p<0.05

**p<0.01

***p<0.001

## Discussion

Patient-level data from a wide range of ART facilities in Kenya, Uganda, and Zambia supports the assertion that national HIV programs have moved quickly to adopt WHO ART first-line treatment recommendations into clinical practice. By 2011, just one year after formal adoption of the revised WHO recommendations, all three countries had shifted away from d4T-based first-line treatment, such that less than 15% of all patients were being prescribed d4T at the time of treatment initiation.

However, the timing and pace of change varied considerably by country. Both Zambia and Uganda moved away from initiating patients on d4T prior to the official release of the 2010 WHO guidelines. In Zambia, national guidelines no longer included d4T-based treatment beginning in 2008.[19] Zambia was also one of the earliest sub-Saharan African countries to adopt TDF, with over a third of initiates receiving TDF as early as 2007. Interestingly, in Uganda, d4T was still included as a recommended first-line treatment option even as the phase-out of d4T was occurring through 2008 and 2009.[20] In contrast to Zambia, Uganda opted for an AZT-based alternative at first, with TDF use only expanding in the more recent initiates. Kenya, on the other hand, waited to update national policy until it was clear that new WHO guidelines would be released recommending phase-out of d4T.[21] The reason for the variation in this shift is likely multifold. First, countries with greater confidence in donor funding may be more willing to adopt more expensive ART medications. Second, countries with a more established epidemic (e.g., South Africa), and therefore more clinical and donor experience around HIV treatment, may be more aggressive in making the switch. Finally, it is likely that while policymakers may respond to early published data (as was the case in Zambia), respected global bodies that synthesize evidence and develop recommendations continue to be important in influencing policy change as well.

In Kenya and Uganda, drug choices varied substantially by facility. In Uganda, we found that patients at private facilities were more likely to be prescribed TDF. This finding likely comes as a result of public facilities following national guidelines that named AZT-based therapy as the preferred first-line therapy in 2010, whereas private facilities likely had more leeway with medication procurement and prescribing patterns.[22] There is also likely to be a significant financial component to this, as public facilities are often underfinanced compared to their private counterparts. The Ugandan government has since revised this to specify TDF-based therapies as the first choice.[23] Ugandan patients were also more likely to be prescribed TDF if there was a doctor on staff at the facility and if a CD4 count was performed. These associations between provider cadre, testing, and drug choice suggest possible relationships between staff training/expertise and following/understanding guidelines and represent a clear opportunity for further investigation as task shifting has become a key component in enhancing medical capacity.[24–26]

It is also notable that many facility factors were not significant predictors of regimen type. Our finding that both hospitals and health centers have similarly moved away from prescribing d4T is contrary to the results of a smaller study from Lesotho that found decentralized health centers to be slower in the adoption of alternative regimens.[27] Additionally, rural facilities performed as well as their urban counterparts when controlling for presence/absence of physician providers. These are encouraging findings given that ongoing scale-up of national ART programs will necessitate increased use of lower-level health centers in order to increase overall ART coverage rates and reach populations with less access to HIV care.

Interestingly, in both Uganda and Kenya, patients classified as WHO stage IV at initiation had over twice the odds of being prescribed a TDF-based therapy as those classified as stage I. This could indicate prioritizing TDF for the sickest patients and may be associated with its higher cost or perceived higher efficacy. Further research examining drug stocks and their relationship to ART prescribing patterns would better help elucidate this concept.[28]

This study benefits from a large and diverse sample in terms of time, geography, and facility type, but it is not without limitations. Despite efforts to sample from all patient charts, facilities use different practices in storing charts of dead or defaulted patients, and this may have differentially affected the sample of charts across facilities. While it is unlikely that charts of patients initiated on a certain regimen were systematically misplaced or removed, it is possible that patients initiated on d4T are actually underestimated given that they are at higher risk of default secondary to medication complications. In addition, we received electronic data records from a small number of facilities, while for the majority of facilities, chart extractions were done by hand; therefore, it is possible that the quality of the data and information included differs for these facilities. However, a separate analyses of these facilities finds that they are within the expected range of prescribing patterns. Furthermore, since charts were weighted based on the size of the ART program in the year of ART initiation, we do not expect that the facilities with electronic records had undue influence on our overall descriptive findings.

In addition to ongoing monitoring of ART prescribing, a key quality metric in evaluating ART programs, we believe that there should be continued attention to the cost implications of d4T phase-out and TDF scale-up. Since the institution of the 2010 WHO recommendations, the cost of both AZT and TDF regimens has decreased, although d4T still remains less expensive. Several studies have examined the potential cost effectiveness of switching to a more expensive TDF-based regimen, taking into account improved adherence, fewer instances of second-line substitution, and overall improved outcomes.[29][30] While the findings are country dependent and vary by the national purchase price of TDF, they report that there may actually be cost savings associated with making such a change. As donor funding for HIV plateaus or declines and the responsibility for financing ART programs slowly moves from donors to countries, it will be important to monitor if low-income countries such as Kenya, Uganda, and Zambia are able to maintain and/or increase ART coverage while utilizing TDF-based treatment regimens.[31–33]
